# Childhood obesity prevention policies in Iran: a policy analysis of agenda-setting using Kingdon’s multiple streams

**DOI:** 10.1186/s12887-021-02731-y

**Published:** 2021-05-27

**Authors:** Shahnaz Taghizadeh, Rahim Khodayari-Zarnaq, Mahdieh Abbasalizad Farhangi

**Affiliations:** 1 Department of Community Nutrition, Faculty of Nutrition, Tabriz University of Medical Sciences, Tabriz, Iran; 2 Department of Health Policy and Management, School of Management and Medical Informatics, Tabriz University of Medical Sciences, Tabriz, Iran; 3 Department of Community Nutrition, Tabriz University of Medical Sciences, 5166614711, Attar Nishabouri St, PO BOX: 14711, Tabriz, I. R Iran

**Keywords:** Childhood obesity, Prevention, Agenda-setting, Kingdon’s multiple streams

## Abstract

**Background:**

Pediatric obesity is one of the most important health challenges of the twenty-first century. Primary prevention of childhood obesity, can lessen its consequences. This study aims to assess childhood obesity prevention policies in Iran through a policy analysis of agenda-setting using Kingdon’s multiple streams.

**Methods:**

A qualitative study was conducted using in-depth interviews with 39 key informants and document review from different stages of the policymaking process of childhood and adolescent obesity prevention programs in Iran. The analysis of documents and interviews were guided based on Kingdon’s multiple streams (problem, policy and political streams).

**Results:**

The important factors of the problem stream were the high prevalence of childhood and adolescent obesity and its risk factors in Iran. In the policy stream, a focus on preventing non-communicable diseases in the health system, increasing the workforce in health centers, promoting health school programs, and creating healthy eating buffets in schools was identified. Under the political stream, the impact of the WHO ECHO program in 2015 and the implementation of the health system transformation plan in Iran in the new government took place after 2013, caused the Iran ECHO program entered the agenda and implemented from 2016.

**Conclusions:**

Now that a window of opportunity for childhood and adolescent obesity prevention policymaking has been created, the problems such as the therapeutic approach in the health system, the existence of sanctions against Iran and outbreak of coronavirus disease-19 (COVID-19), have hindered the successful implementation of this policy and the opportunity window has not been well used. However, actors need political support from the high levels of government to keep this policy on the agenda.

## Background

Childhood and adolescent obesity (CAO) is one of the most serious risk factors for cardiovascular and metabolic diseases [1]. The world health organization (WHO) reports over 340 million children and adolescents aged 5–19 were overweight or obese in 2016 [2] and the rate of CAO in developing countries is 30% higher than in developed countries [3]. Although inactivity and intake of high energy foods are the main causes of CAO, studies show that nutritional transition, stunting, and intake of poor micronutrient foods in developing countries can be major challenges [4, 5]. It is noteworthy that the Asian race is more vulnerable to body mass index (BMI) increase than Western races, so, a slight increase in BMI compared to normal in the Asian race can show higher complication [6]. The results of CASPIAN-V (Childhood and Adolescence Surveillance and Prevention of Adult Non-Communicable Disease) study of 14,118 subjects aged 7 to 18 years of 30 provinces in Iran, showed that the prevalence of overweight and obesity were 9.4 and 11.4% respectively [7]. Heshmat et al. also, showed that 58% of children and adolescents in the CASPIAN-V study had low physical activity and adherence of healthy nutritional behaviors in 53.6% of them was low or moderate [8]. WHO, advise governments to implement population-based strategies to combat obesity [9]. Review studies show that different CAOP policies were adopted in each society based on their structure [10], however, in 2015, WHO, in collaboration with 100 member countries, launched a program called Ending Childhood Obesity (ECHO), and persuaded member countries to implement the program to prevent and treat CAO [11]. Based on this, we decided to examine how and when the CAO prevention policies were on the agenda, before and after the ECHO program. In this regard, we used the Kingdon’s multiple streams framework.

### Conceptual framework

**Fig. 1:**
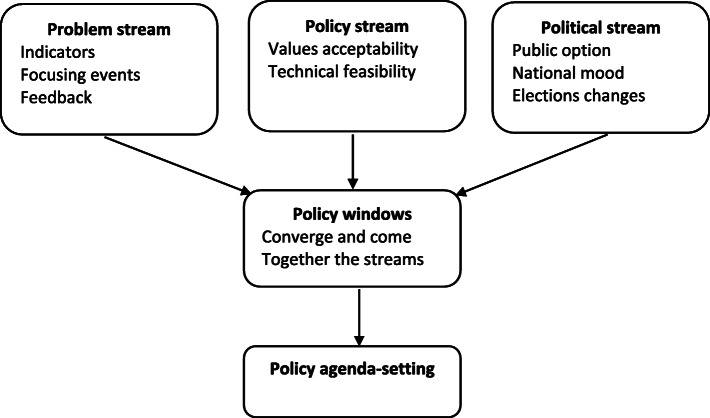
The Kingdon’s multiple streams framework

Agenda setting is part of the policy process proposed by Mirzoev [12] and Fafard [13] which is an important part of policy analysis. This approach allows researchers as well as policymakers to divide the policy-making process into specific stages, each of which has a distinct content and structural features, to better study the process. Kingdon was the first to suggest dividing policy-making into separate sets or streams [14]. The Kingdon model focuses on the role of key policy stakeholders inside and outside the government, which seizes streams to create opportunities called “policy windows”. This framework determines what were the problem streams? What were the policy streams? What was the politics stream at that time? And finally, what made the opportunity window open? The problem stream refers to policy reports, statistical indicators, and pressure from the advocacy groups which attract policymaker’s attention to that problem. The policy stream explains the solutions developed for a situation, and the political stream clarifies the political factors whether or not an issue emerges on the agenda. According to Kingdon’s model, these three streams operate separately and in independent channels, but, when all of the streams converged and come together the “window of opportunity,” will be created [14] Fig. 1.

Previous researchers have also used the Kingdon’s multiple streams framework to examine the inclusion of health policies on the agenda. Craig et al., used this framework to figure out how Arkansas’s Act 1220, school-based childhood obesity prevention policy, were put on the agenda and documented factors that produced a policy window and allowed entrepreneurs to enact comprehensive legislation [15]. Sainsbury et al. also, demonstrated that, despite significant evidence of the impact of the SSB tax on reducing the prevalence of obesity and existence of opportunities for policy reform in Australia over the past 30 years, an incomplete joining of the problem, policy and politics streams due to inadequate pressure for change from civil society, fragmented public health advocacy efforts, industry interference, and pragmatic political agendas has prevented a SSB tax policy window from opening [16].

## Methods

Using Kingdon’s agenda-setting framework, this qualitative study explores the principal factors influencing the problem stream of CAO, the solutions to address CAO, and the political events affecting the emergence of childhood and adolescent obesity prevention (CAOP) policies on the agenda.

### Document review and key informant interviews

This study was conducted adhering to a qualitative-descriptive research design which was part of large-scale research entitled “Future Studies and Analysis of Childhood and Adolescent Obesity Prevention Policies in Iran and Presenting Policy Options”. Accordingly, documents from the searching databases of Google Scholar, PubMed and SCOPUS as well as the websites were reviewed. Then, using a semi-structured interview and a snowball sampling method, after informing them of the objectives of the study, were interviewed until data saturation was reached.

The interviewer tried to avoid any bias or prejudice during the interview. Field notes were made during the interview and to complete the text of the interview, participants were asked to record their voices during the interview if they were satisfied. Transcripts returned to participants for comment and/or correction. The comments from the semi-structured interviews were coded by the researcher and the obtained codes were classified together according to their similarity to form themes and sub-themes. We tried to include stakeholders of different CAOP sectors in policymaking. The interviews took place between November 2019 and March 2020. Thirty-four interviews were conducted face-to-face and 5 of them by telephone, following informed consent. We used a topic guide that was designed based on Kingdon’s multiple streams theory. The topic guide was pre-tested in three interviews and based on the results from the analysis, some changes were made to the topic guide. The following questions were asked:

1. Problem stream: What are the unresolved issues in the field of CAOP in Iran?
2. Policy stream: So far, what solutions have been adopted by various organizations to address CAOP-related issues in Iran?
3. Political stream: What are the political factors that have an impact on CAOP prevention policies in Iran? Is there political support form the highest level of government for CAOP policymaking in Iran?

### Data analysis

The conventional content analysis was used to analyze data through MAXQDA software (version, 2010). All collected materials were open-coded independently by two authors and categorized by the similarity of their content. The difficulties in coding and categorizing were discussed by the two authors (ShT and RK). After data analysis, 412 codes emerged related to the CAOP policies that after reviewing the similarities and merging of the codes, they were reduced to 125 codes and categories developed. After linking the underlying meanings of categories using inductive analysis, themes emerged

### Rigor of the study

We tried to include stakeholders of different CAOP sectors of policymaking to increase the conformability of the study. Inter-observer reliability was used, and disagreements were resolved through discussion. Also, peer check was done by sharing extracted themes with other co-author (MAF). A member check was done after each interview, showing the notes and asking what was understood from interviewees. We collected and analyzed the data simultaneously.

### Ethics and consent

This work was supported by Tabriz University of Medical Sciences (Grant number: 62918).

And was approved by the Ethics Committee of Tabriz University of Medical Sciences (IR.TBZMED.REC.1398.840). Signed informed consent was obtained from the key informants at the beginning of the interview. Also, interviewees were free to withdraw from the study for any reason at any time. The present study aimed to evaluate the possibility of policy change opportunity regarding childhood and adolescent obesity prevention (CAOP) through Kingdon’s agenda-setting framework.

## Results

Twenty policy documents (Table 1) were reviewed and 39 key informants, with an average age of 44.76 years old and 15.47 years of work experience, after informing them of the objectives of the study, were interviewed (Table 2). The average interviews time was about 1 h (59.6 min). The results of the review of documents as well as the interview are explained in the form of Kingdon streams.

**Table 1:** List of analyzed policy documents

No	Document	Publisher organization	Year of publication
1	Optimal food basket for Iranian society	MoHME	2013
2	WHO Ending Childhood Obesity	WHO	2015
3	Amendment of the executive instructions of the PHC expansion plan for the realization of UHC in urban areas for 2018	MoHME	2018
4	Special nutrition education package for a health care provider in the health transformation plan in the field of health	MoHME	2019
5	Healthy instructions for healthy nutrition buffets in schools	MoHME	2013
6	assessing the micronutrient status in Iran in 2012	MoHME	2012
7	Executive instructions of health-promoting schools in the Islamic Republic of Iran	MoE	2010
8	Executive instructions of the Health Ambassadors Program for the academic year 2017–2018	MoE	2018
9	Instructions for a comprehensive program to prevent and control overweight and obesity in children and adolescents	MoHME	2016
10	Instructions for providing a hot meal in the rural kindergarten	Welfare Organization	2007
11	Executive instructions for in-school and out-of-school sports centers	MoE	2015
12	Guideline for caring for and managing the weight of children under 5 years of age, for health care providers in the country’s health system	MoHME	2018
13	Health culture promotion program document approved by the Public Culture Council of 2018	Ministry of Culture and Islamic Guidance	2018
14	Fundamental Transformation Document of the Ministry of Education	MoE	2011
15	National Document for the Prevention and Control of Non-Communicable Diseases and Related Risk Factors in the Period 2015–2025	MoHME	2015
16	A set of basic interventions for non-communicable diseases in the Iranian primary health care system “IRAPEN”	MoHME	2017
17	National Document on Nutrition and Food Safety 1399–1391	MoHME	2012
18	Action plan for performing stretching exercises in the classroom, a solution to promote optimal physical activity in the community	MoE	2014
19	Care system for prevention of behaviors and risk factors for students’ health (CASPIAN Studies 1–5)	MoHME	2003–2014
20	Comprehensive scientific map of the country’s health	MoHME	2010

*MoHME* Ministry of Health and Medical Education, *MoE* Ministry of Education, *WHO* World Health Organization

**Table 2:** The characteristics of participations in the study

Stakeholder’s name	Participant’s No	work experience (year)	Age (year)	Level	Sector	Related role
Department of education (Professor of the University of medical sciences of MoHME)^a^	P1	12	40	Regional	Professional	Collaborate in developing policies to prevent childhood obesity by nutrition education in the university
Welfare Organization	P2	12	39	National	Governmental	Policymaking for nutrition education to children and parents and adherence to the principles of healthy nutrition in kindergartens and childcare centers
Municipality	P3	8	49	Regional	Non-Governmental	Providing sports equipment and spaces for children and adolescents
Ministry of Sports and Youth	P4	15	51	Regional	Governmental	Providing appropriate facilities for exercise in childhood
Imam Khomeini Relief Committee	P5	10	58	Regional	Non-Governmental	Providing healthy food items for financial trouble families as well as education healthy lifestyle in family training sessions
Islamic Development Organization	P6	15	41	Regional	Governmental	Advertising of nutrition and PA in the community
Secretariat of the Health and Food Safety	P7	3	41	Regional	Governmental	Establish better coordination and communication between stakeholders and organizations
Health care providers of MoHME	P8	20	52	Regional	Governmental	Collaborate on primary health care, nutrition education and PA advice screening for overweight and obese children and adolescents in health centers
Student Organization	P9	2	40	Regional	Non-Governmental	Collaborate on providing school buffet snakes
Food and Drug Office	P10	18	52	Regional	Governmental	Increase the level of safety of food and beverages
School Health Department of the Province’s Health Center	P11	24	55	Regional	Governmental	Coordinate with schools to provide nutrition and PA training and screening overweight and obese children and adolescents
Food and Drug Organization	P12	17	40	National	Governmental	Policymaking to increase the level of safety of food and beverages
Children and Adolescents’ Intellectual Development	P13	20	49	Regional	Governmental	Participation in healthy nutrition education for children and adolescents
Welfare Organization	P14	27	50	Regional	Governmental	Collaborate on nutrition education for children and parents in kindergartens and observing the principles of healthy nutrition in kindergarten food and child care centers
Population and Family Health Department [1]	P15	9	53	Regional	Governmental	Collaborate on primary health care and screening for overweight and obese children and adolescents in health centers
Ministry of Education	P16	12	39	National	Governmental	Collaborate with the MoHME in develop or implement the nutrition education, school foods or PA^a^ policies in schools
Department of education (Professor of the University of medical sciences of MoHME)	P17	6	59	National	Governmental	Cooperation with the MoHME and WHO in promoting Iran ECHO, Responsible for implementing Iran ECHO pilot program in Isfahan (one of the cities in Iran)
The Office of Community Nutrition Improvement [1]	P18	10	48	National	Governmental	Mainly responsible for the CAOP^b^: Policymaking, design and implementation of nutritional interventions
Islamic Republic of Iran Broadcasting (IRIB)	P19	12	45	National	Media	Producing educational and informational programs as well as harmful food ads
Health Deputy of MoHME	P20	15	58	National	Governmental	Policymaking for health service system in Iran
Executive manager in the Ministry of Education [1]	P21	25	52	Regional	Governmental	Collaborate in implementation of the nutrition education, and PA policies in schools
The office of the Community Nutrition Improvement department of the Health Center	P22	15	40	Regional	Governmental	Policymaking, design and implementation of nutritional interventions at the provincial level
School Health Department of MoHME	P23	30	50	National	Governmental	Policymaking for nutrition education, providing healthy food, as well as training and doing PA in schools
Population and Family Health Department [2]	P25	15	40	Regional	Governmental	Collaborate on primary health care and screening for overweight and obese children and adolescents in health centers
Non-communicable diseases Department of the Province’s Health Center	P26	12	39	Regional	Governmental	Collaborate on the development of guidelines for non-communicable diseases prevention
Population and Family Health Department [3]	P27	23	45	Regional	Governmental	Collaborate on primary health care and screening for overweight and obese children and adolescents in health centers
Boys’ high school physical education teacher	P28	16	42	Regional	Governmental	Collaborate in implementation of the PA policies in schools
The headmaster of boys’ high school [1]	P29	25	48	Regional	Governmental	Collaborate in implementing CAOP policies in schools
The headmaster of boys’ high school [2]	P30	28	47	Regional	Governmental	Collaborate in implementing CAOP policies in schools
Girls’ high school physical education teacher [1]	P31	30	51	Regional	Governmental	Collaborate in implementation of the PA policies in schools
Girls’ high school physical education teacher [2]	P32	15	37	Regional	Governmental	Collaborate in implementation of the PA policies in schools
The headmaster of girls’ high school [1]	P33	27	48	Regional	Governmental	Collaborate in implementing CAOP policies in schools
The headmaster of girls’ high school [2]	P34	13	37	Regional	Governmental	Collaborate in implementing CAOP policies in schools
Executive manager in the Ministry of Education [2]	P35	24	47	Regional	Governmental	Collaborate in implementation of the nutrition education, and PA policies in schools
The Office of Community Nutrition Improvement [2]	P36	18	44	National	Governmental	Mainly responsible for the CAOP: Policymaking, design and implementation of nutritional interventions
Office of the Parents and Teachers Association of the Ministry of Education	P37	4	40	Regional	Governmental	Collaborate on communication between parents and school officials
Executive manager in the Ministry of Education [3]	P38	8	48	Regional	Governmental	Collaborate in implementation of the nutrition education, and PA policies in schools
Manager of food production and processing factory (cakes and cookies)	P39	5	43	Regional	Private	Participation in the production of a variety of food products

^a^ *MoHME* Ministry of Health and Medical Education

^b^ *CAOP* childhood and adolescent’s obesity prevention

### Problem stream

#### High prevalence of childhood and adolescent obesity in Iran

Studies show an upward trend in the prevalence of CAO in Iran. These studies also show that obesity prevention activities in this age group have many gaps and barriers and have not been able to control this disease [17, 18]. It has been reported that the prevalence of obesity was 8% in children under 5 years in 2019 [19], 13.1% in boys and 9.8% in girls, in the age group of 5–9 years, and 9.3% in boys and 8.1% in girls in the age group of 10–19 years in 2016 [18].

#### High prevalence of childhood and adolescent obesity risk factors in Iran

The main causes of CAO (inactivity and high energy intake) and some of their risk factors such as knowledge and perception of weight, socioeconomic status, fast food advertising, family history of obesity, duration of exclusive breastfeeding, do not have a favorable situation in Iran [17, 20–22]. In 2016, WHO statistics related to Iran’s health profile showed that only 23% of infants under 6 months were exclusively breastfed [23], which indicates inappropriate nutrition in the first 6 months of life. One of the main reasons affecting food intake in infancy, childhood and adolescence is insufficient parental awareness of obesity and its consequences. One stakeholder states:

“*Student control is not entirely in the authority of schools, therefore, our main problem and obstacle is parents who have low nutritional awareness*" (p.21).

Also, one of the Welfare Organization stakeholders explains socioeconomic status as the reason for the problems in implementing CAOP policies in kindergartens” *.... Even if there is a policy, due to lack of budget and economic problems in our organization, we cannot do any policy in this regard* “(P.14).

Also, high access to fast food is an important problem in Iran. One of the stakeholders says in this regard: “*Even if schools follow the rules because the stores near the school also sell unhealthy food, then the policies will be difficult*” (p.33).

### Policy stream

Attention to the prevention of CAO occurred after a national study of micronutrients in 2001 as “assessing the micronutrient status in Iran”. In that year, according to WHO criteria, 29.1% of 15–23 months’ children and 10% of 6 years old children were overweight and obese. So, the ephemeral CAOP practices emerged after that study. A review of texts, websites and the results of interviews showed that the following activities were performed as a solution to reduce obesity in children and adolescents: nutrition and physical activity (PA) education (in kindergartens, schools, health centers and in the community), improving the status of primary health care, controlling sugar and fat levels and use of food labels and lights on the produced foods, monitoring of healthy nutrition bases of school buffets, health-promoting schools plan and dynamic schools plan. The results of a study in 2012 showed that those interventions failed to control obesity among children and adolescents [24].

The interventions in this regard can be examined in two parts:

A. *Under 6 years old children:*

Except for two studies cited in 2001 and 2012 that have checked 15–23 months’ children, no other nationwide study looked at the prevalence of obesity in children under 6 years of age, but, some strategies and interventions have been done in this regard.

The results of the present study showed that many factors are the main barriers to exclusive breastfeeding of neonates in Iran; such as the high availability of infant formula, the recommendation of pediatricians to use infant formulas, encouraging by relatives to use sugar water, cow’s milk, formula or start complementary feeding earlier.

“*Parents often ask health workers to prescribe infant formula, and if our colleagues, pediatricians will easily prescribe that. If you can change the minds of pediatricians and gynecologist who do not prescribe infant formula indiscriminately, we can get good results in the long run*” (P.25).

Among the solutions adopted in this section for children under 6 years of age, was the development of guidelines in education for nutrition, exclusive breastfeeding, proper complementary feeding, increase the PA for pregnant and lactating mothers and mothers with children under 6 years of age in health centers and children in the kindergartens. Due to a lack of manpower time, the implementation of nutritional education interventions faces many challenges.

"One of the stakeholders of the Welfare Organization in the policymaking level, says "*Benefiting from the expertise of our organization and the participation of other agencies such as the Nutrition Improvement Office of the Ministry of Health, the target community is educated and informed about nutrition and PA* (P.2).

Also, the establishment of child-friendly hospitals since 1992 in most hospitals with gynecology and obstetrics, NICU and pediatrics units, and the promotion of breastfeeding through this program, is one of the activities taken in this area.

B. *Children and adolescents 6–18 years:*

With the beginning of the weekly iron supplementation program, for female students in middle and high school, since 2004, it was suggested that students should receive nutrition education simultaneously with supplementation programs. The result of this study showed that nutrition education was not successful in that program.

“*Implementing nutrition education along with iron supplementation has many problems because there is not enough time and opportunity to do these training in schools and it takes time for classes*” (P.32).

The next strategy was the increase of nutritionists staffing number in health centers since 2014, however, due to the interference of school time with the working hours of nutritionists in health centers, there are many problems in the implementation of nutritionists services for students in health centers.

one of the key informants in this regard says “... *but because there is not enough opportunity for the health staff, so we have to be content with a few general recommendations and we cannot do all the training in detail based on the system*.” (P.8).

Other programs included creating a dynamic schools plan in some schools to increase PA.

### Political stream

The simultaneity of the first national food security survey in 2011, formation of a specialized working group on health and food safety in 2012, formation of a national document on health and food security, as well as a national document on prevention of non-communicable diseases and publication of CASPIAN- V study results in 2014, increased policymakers’ attention to CAO and government support with the launch of Iranian health care system reform plan, opened the policy windows to perform the proposed program by WHO (ECHO), with the responsibility of the Nutrition Improvement Office of the Ministry of Health. One interviewee working with MoHME explained that:

” *after the health transformation plan, two important things happened that are unique: one is the use of nutrition experts and the other is that the programs were integrated with the view of preventing Noncommunicable diseases (NCD), obesity and overweight*” (P.18).

Accordingly, the way was paved for political support for the actions of various stakeholders involved in the prevention of CAO. So, since 2016, with the implementation of the Iran ECHO (IRAN-Ending Childhood Obesity) program, this plan has been implemented in some provinces of Iran [25].

When deciding to adopt a solution, the prevention approach is often considered, but in the implementation phase, more attention is paid to treatment than prevention in the health system.

"*In macro-level policies ... we have to move towards prevention. ... But this is something that most of our government officials do not have the right interest in and attitude towards*," said one source (P.21).

## Discussion

The key findings indicated that the major problems were the lack of cooperation and coordination between stakeholders, the priority of the treatment approach over prevention in the health system, economic problems and low awareness and attitude towards CAO as an obstacle to the agenda-setting of this policy or not implementing the policy optimally after it was put on the agenda.

For a health-related issue to appear on the agenda, the policymaker needs to provide a source of evidence to highlight this issue [26]. In our study, the impact of statistics on the problem of CAOP policies was also important because it was one of the important components of evidence that required action from stakeholders. A major concern in this area, is the high prevalence of CAO, despite numerous actions taken by the Ministry of Health and Medical Education (MoHME) and some other stakeholders, before and after the implementation of the Iran ECHO program [18, 19]. The studies in other countries, also, showed that despite some obesity prevention policies, the prevalence of obesity in this age group not controlled [27–29]. The switch in lifestyles from traditional to western type, which has exacerbated the problem of sedentary lifestyle and intake of high-energy, low-nutrient foods, is the second major indicator that highlights the issue of CAOP policies for policymakers [30].

The results of the studies in Iran, which are consistent with studies done in most other countries such as Vietnam and China [31, 32] showed that CAO is more prevalent in urban areas than in the rural areas and in boys higher than girls [17, 18]. In the urban areas processed and fast foods are more available, the game shops for children and adolescents are more common and spaces for play are more limited than in rural areas. So, the differences in obesity prevalence between the urban and rural areas were due to differences in community characteristics [32]. Studies demonstrated that girls and boys differ in environmental factors (hormones, ethnicity, weight gain patterns, susceptibility to social factors, and body composition) and genetic, which may lead to gender differences in the prevalence of obesity [33, 34]. Despite the high evidence of a higher prevalence of obesity in boys than girls, policies do not pay more attention to obesity in boys, and even nutrition education in schools, along with iron supplementation programs, is done only for female students, which requires revision in policymaking. The first CASPIAN study in 2003 showed that inactivity and unhealthy nutrition were prevalent among children and adolescents [35]. So that, Rahmanian et al. 7 years later, in a study showed that the policies in that period, has not improved the obesity prevalence problem, due to the nutrition transition and rapid lifestyle changes. They showed that the prevalence of obesity and overweight in children and adolescents aged 7–18 years reached from 4.5% and 8.2 in 2003 to 7.5 and 9.1% in 2010, respectively [30].

Children and adolescents, were exposed to widespread marketing of unhealthy foods in Iran [22]. Considering that more than 60% of TV commercials are related to food in Iran [36] and most of these commercials are related to unhealthy foods [37].

Other factors which affecting CAO, were the misperception of weight by children, adolescents, parents or even school officials. So that, the national study in 2014 among 14,440 children and adolescents aged 18–7 years showed that misperception of weight is very common among Iranian children and adolescents and its rate among children and adolescents was 59.1% which, 30.8% of the participants reported underestimated weight [20]. Most of the interventions and solutions adopted in Iran are in the form of nutrition education and less intervention is done to increase physical activity. As the results of the interviews with stakeholders showed that due to the high workload of teachers and insufficient manpower of the health care sector in schools, nutrition education and PA training are not fully done. Although the guidelines developed by the Ministry of Education emphasize the increase of physical activity, the results of studies showed that PA in schools is less than the recommended values [38]. A study in 2018 by Wang et al. found that implementing a policy of increasing 1 h of PA per week more than routine time, combined with 24 theory sessions for 1 year, could control the prevalence of CAO. In that study, the prevalence of obesity at the beginning of the intervention was 16.6%, which after 1 year reached 16.9% in the intervention group and 18.6% in the control group [39]. Also, nutrition education without changing the structure of society will not lead to effective results. High access of children and adolescents to unhealthy food in stores near the schools and sometimes in the schools at low prices, can be one of the important structural factors in this area in Iran. In 2017, the MoHME developed IraPEN (Package of Essential Non-Communicable (PEN) Disease Interventions for Primary Health Care in Iran) program to control NCDs [26], but unfortunately, this program does not pay much attention to children and adolescents.

In the CAOP related programs in Iran, before and after the Iran ECHO, despite considering both treatment and prevention in the formulation and development level, in the implementation level, the prevention aspect is neglected. More focus on curative care than prevention in this age group in the health care system is one of the issues to be considered in the policies adopted by the stakeholders of the health system. Doshmangir et al., also, demonstrated that the Iranian government was more focused on curative care, while, less attention was given to public health and preventive interventions [40]. Except for reducing the sugar of carbonated beverages and fats of some food products and use of food labels and lights on the produced foods, that benefit all age groups in society [41], other community-based policies, such as, increasing sports equipment in the cities, increasing community based educational programs by the media, and municipalities education centers pays more attention to adult obesity.

Our study highlights the impact of international support and national political content in shaping the agenda for CAOP policies. In 2015, WHO formed a committee to end the childhood obesity in the world (ECHO) program. The program includes strategies that should be implemented with the participation of all sectors and stakeholders to develop the policy. The implementation of the health system transformation plan in Iran in the new government took place after 2013 and more attention was paid to NCD control in this plan [40]. Therefore, the change of government in 2013 was another important political factor that caused the Iran ECHO program entered the agenda and implemented in 2016. The effects of change of government and its effect on health system policies in the Kabiri et al. study are similar to our study [26].

It was expected that by opening this window of opportunity, the policy flow would be strengthened and the solutions and practical initiatives would be used in this field, but, as the results of the current study showed, the stakeholders other than the health system didn’t show much motivation to participate in the policy. as Haghi et al. stated, CAO is described as very worrying and requires immediate action in Iran [42]. Despite the confluence of three streams at a specific point in 2016, this confluence again distances over time. Numerous factors such as the centralized health policy and the existence of a top-down approach can be effective in not achieving the goals of this policy, which was also observed in the study of Hendriks et al. [43] in top-down policies that are organized at three levels of country, province and city in Iran [44], there is not much consultation of executive stakeholders in the policymaking. When top policymakers do not engage with down executive and local stakeholders, the desired result is often not achieved and may lead to the destruction of initiatives and policies [45]. However, the centralized health system cannot be explicitly described as an inefficient approach in the health system. For example, Cuba has one of the most effective and efficient health systems in the world. Cuba is one of the countries where the health system is central. The characteristics of free public access and the integration of health services as well as the emphasis on prevention services in Cuba are among the most prominent features of Cuban health policies [46, 47].

Another noteworthy point is that economic sanctions against Iran have jeopardized food security, which, by reducing access to healthier food at a higher price, does not move the policy in line with policies adopted to prevent CAO [48]. As mentioned, the launch of the Iran ECHO program could be an important turning point in preventing CAO. Although, the beginning of the activities of this policy, coincided with the economic sanctions and with the outbreak of coronavirus disease-19 (COVID-19) in 2020 [49], it was not possible to implement the policy actively. Studies show that there are conflicts of interest among stakeholders in the health care system and that health priorities are not prioritized properly in Iran [40]. Instability in the MoHME programs as the main stakeholder of CAOP policies, ineffective use of the power of multiple stakeholders and insufficient coordination between them, has led to inadequate development and implementation of CAOP policies in Iran [50]. Although it was thought that the launch of the Iran ECHO program could be effective in CAOP, the policymakers’ attention to barriers of CAOP policies, with priority at the structural level and examining the factors involved in the agenda-setting which led the upward trend of prevalence of overweight and obesity in this age group after 5 years, requires a comprehensive review.

In our opinion, our study is the first study that seeks to identify leverage points to advance the implementation of CAOP policies in Iran, examines all its related policies and describes the barriers and facilitators of their entry into the policy window in Iran. One of the main limitations of the present study was the lack of cooperation of some stakeholders due to the COVID-19 pandemic, which is expected to be overcome in future studies by other researchers.

## Conclusion

This paper provides insights into the positive and negative impact drivers behind the political process of CAOP agenda setting, which can lead to the introduction of a policy package in this issue. Now that a window of opportunity for childhood and adolescent obesity prevention policymaking has been created, the problems such as the therapeutic approach in the health system, the existence of sanctions against Iran and outbreak of coronavirus disease-19 (COVID-19), have hindered the successful implementation of this policy and the opportunity window has not been well used. However, actors need support from the high levels of government to keep this policy on the agenda. Policymakers need to know that the strengthening intra-sectoral cooperation and create advocacy for inter-sectoral cooperation is essential to implementing such policies. The existence of health indicators and the active presence of actors in this period can put pressure on policy change. However, actors need political support to put this issue on the agenda. This support can go beyond organizations such as the MoHME as well as the Ministry of Education to overcome not only political opposition but also actors who were crucial in the implementation phase. It is certain that the findings of this study alone cannot stop the increase in the prevalence of obesity in Iran, but they can be used by decision makers and policymakers in policy decisions by recognizing the facilitators of flows into the opportunity window and the obstacles that prevent better use of this opportunities.

## Data Availability

All data generated or analyzed during this study are included in this published article.

## Abbreviations

BMI: Body Mass Index
COVID-19: Coronavirus disease-19
CAO: Childhood and adolescent obesity
CASPIAN-V: Childhood and Adolescence Surveillance and Prevention of Adult Non-Communicable Disease
CAOP: Childhood and adolescent obesity prevention
ECHO: Ending Childhood Obesity
IraPEN: Package of Essential Non-Communicable (PEN) Disease Interventions for Primary Health Care in Iran
MoHME: Ministry of Health and Medical Education
MoE: Ministry of Education
NCD: Noncommunicable diseases
WHO: World Health Organization
